# Foaming of PCL-Based Composites Using scCO_2_: Structure and Physical Properties

**DOI:** 10.3390/ma15031169

**Published:** 2022-02-03

**Authors:** Katarzyna Kosowska, Jan Krzysztoforski, Marek Henczka

**Affiliations:** Faculty of Chemical and Process Engineering, Warsaw University of Technology, Waryńskiego 1, 00-645 Warsaw, Poland; jan.krzysztoforski@pw.edu.pl (J.K.); marek.henczka@pw.edu.pl (M.H.)

**Keywords:** tissue engineering, supercritical carbon dioxide, foaming, poly(ε-caprolactone), porogen, scaffold

## Abstract

The process of foaming poly(caprolactone)-based composites using supercritical carbon dioxide was analyzed. The impact of the conditions of the solid-foam production process on the process efficiency and properties of porous structures was investigated. The novel application of various types of porogens—hydroxyapatite, nanocellulose, carboxymethylcellulose, and graphene oxide—was tested in order to modify the properties and improve the quality of solid foams, increasing their usefulness in specialized practical applications. The study showed a significant influence of the foaming process conditions on the properties of solid foams. The optimal process parameters were determined to be pressure 18 MPa, temperature 70 °C, and time 1 h in order to obtain structures with appropriate properties for applications in biomedical engineering, and the most promising material for their production was selected: a composite containing 5% hydroxyapatite or 0.2% graphene oxide.

## 1. Introduction

Polymer foams are multiphase materials in which the continuous phase is a dense polymer matrix surrounding gas bubbles. The possibility of combining the properties of both phases has become a promising tool in the field of materials science and industry [1,2,3,4,5]. Depending on their application, some foams exhibit considerable elasticity or brittleness, a high porosity level, and a wide distribution of pore diameters [6].

Recently, researchers have been working on the development of technology for the production of biodegradable foams and environmentally friendly methods for the preparation of functional three-dimensional porous structures that could be successfully used in a wide variety of industries, including as tools for acoustic or thermal insulation, or as scaffolds for cell cultures for tissue engineering [1,7].

At present, many methods of producing functional porous structures are known. Among them, solvent casting and particle leaching, freeze-drying, thermally induced phase separation, powder-forming, sol-gel, and electrospinning are of great importance [8,9,10,11,12,13,14,15,16,17,18,19,20,21,22,23,24,25]. The use of these methods is associated with many advantages, such as the relatively simple operation of the research equipment and the low cost of producing the final porous structure, but also involves certain restrictions, such as the need to use a large amount of harmful volatile organic solvents or the inability to use thermolabile biological compounds due to requirement for high temperatures [10,11,13,21]. Given the above limitations on production methods, special attention was paid to the foaming process using media in the supercritical state [9,26,27,28,29,30,31,32,33,34]. It is worth mentioning that it is possible to modify the characteristics of the produced porous structures by combining different manufacturing methods and using composite materials [35,36,37,38,39,40,41]. Additionally, the morphology of the polymer foam is modeled by the properties of the polymer and its structure, crystallinity and degree of hardening [42]. Recently, polymer nanocomposites have gained considerable popularity as materials of high utility. In the work of Thanh et al., a promising polymer nanocomposite with improved properties was obtained, as a modified clay was added to the polyester [43]. In the next work, the necessity to analyze the strength and stability of the obtained composite materials was emphasized [44]. There are also many scientific reports in the literature on the adaptation of the manufacturing process to obtain porous structures with the appropriate properties. An ideal summary of the considerations concerning the influence of the mold shape on the foam properties was made in [45].

Generally, the process of foaming polymers using supercritical carbon dioxide is based on three basic steps [27,29,35,36,46,47]. The first step consists in saturating the polymer material with CO_2_ at an elevated pressure and temperature. As a result of this process, the material plasticizes and swells, and its glass transition temperature, viscosity and surface tension decrease. Gas diffusion creates a homogeneous polymer/CO_2_ mixture. The next steps involve system cooling (step II) and rapid decompression (step III), resulting in a shift in the thermodynamic equilibrium of the mixture, as well as the nucleation and growth of gas bubbles in the matrix of the polymer being processed. As a consequence, the amount of carbon dioxide present in the polymer material will decrease, and the glass transition temperature of the polymer will increase again. At some point, the polymer returns to the glassy state, the solid foam solidifies, and the growth of pores inside the structure is stopped. The pressure and saturation temperature of the CO_2_ polymer material and the expansion rate of the system largely determine the morphology of the resulting solid foam.

Currently, solid foams are commonly produced using the method of foaming materials using supercritical media with varying efficiencies. Xu and Huang [48] studied the process of expanding polystyrene, resulting in solid foams with a bimodal pore-size distribution. It was demonstrated in the paper that by regulating the process parameters one can control the properties of the obtained structures. Mi et al. [49] dealt with the creation of PCL and CNC composite structures using the extrusion method using scCO_2_. In their work, a significant impact of the additive on the performance properties of the obtained products was observed. Chen et al. [33] investigated the impact of polymer-foaming performance parameters with two-stage system decompression. Promising porous structures were used in tissue engineering for bone regeneration [50]. Baldino et al. demonstrated the high efficiency of the use of supercritical media to obtain dry porous structures in a short time, emphasizing the possibility of regulating the morphology features by changing the parameters of the manufacturing process [51]. Many studies have demonstrated the effect of the addition of additive nanoparticles as additional nucleation sites, i.e., the decrease in the size of the pores and the increase in the pores density of the resulting porous structures [52,53]. In the context of specific applications, it seems important that the solid foam meets the given requirements regarding its morphology, mechanical and biological properties. In the literature on the subject, there have been many reports on particularly important parameters which determine the usefulness of porous structures for the purposes of biomedical engineering. The morphology properties are assessed on the basis of the porosity and pore size, number and distribution in the solid foam. Various applications require a very wide range of variability of the required pore diameters for the development of one type of cells. The optimal values of the mean pore size of structures which are useful in tissue engineering can be summarized as follows: 5 μm pores are required for newly formed blood vessels, 5–15 μm pores are required for connective tissue cells in the growth phase, roughly 20 μm pores are required for hepatocytes, 20–125 µm pores are required for the skin regeneration process in an adult humans, and less than 100 to 450 µm pores are required for bone tissue regeneration; fibrous cartilage prefers both macropores (150–300 µm) and micropores below 50 µm [19,54,55,56,57,58]. The porosity of a scaffold with high utility as a bone implant should be around 80%, but at the same time there should be a balance between the rate of degradation and the mechanical properties [39,56,59,60,61,62]. When analyzing the mechanical properties, stiffness and mechanical strength are very important, and the values of these parameters differ significantly depending on the place of the implant’s application. For example, for bone tissue, the compact body is characterized by a Young’s modulus in the range of 7–30 GPa and a mechanical strength of 100–230 MPa, while the spongy body, which is much more porous, has a Young’s modulus of 0.02–0.8 GPa and a compression of 2–12 MPa [60,63,64]. The differences in these sizes result from the structure of the bone, which has crystals of minerals and collagen arranged in the axial direction, thus ensuring greater mechanical strength [63,64]. Its biological properties make the material non-toxic to cells and thus biocompatible [5,14,60].

Therefore, the main goals of this study are to investigate the process of foaming materials using supercritical carbon dioxide, to gain knowledge about the mechanism of the foaming process using scCO_2_, and to identify the influence of the foaming process parameters of saturation pressure, saturation temperature and saturation time on the properties of the resulting porous structures. Moreover, it is important to investigate the possibility of a new application of various types of pore-forming substances, such as hydroxyapatite, cellulose, carboxymethylcellulose, graphene oxide, and their mixtures as raw materials in the production of useful solid foams, and to prove the beneficial effect of their use on the course of the poly(ε-caprolactone)-foaming process using supercritical carbon dioxide, as well as the effective modification of the properties and the improvement of the quality of the produced solid foams, increasing the usability of the produced porous materials in specialized practical applications in tissue engineering.

The specific objectives of this paper are:

1. the experimental determination of the effect of the conditions for the implementation of the poly(ε-caprolactone)-foaming process without the addition of blowing agents on the properties of the produced solid foams;
2. the experimental determination of the effect of the concentration, particle size and type of pore-forming substances on the functional properties of the produced porous structures;
3. the assessment of the usability for tissue engineering applications of solid poly(ε-caprolactone) foams produced in the foaming process using pore-forming substances—hydroxyapatite, cellulose and carboxymethylcellulose, as well as graphene oxide—and the identification of process parameters with key impacts on the performance of these materials.

## 2. Materials and Methods

### 2.1. Materials

Experimental studies of the process of polymer material foaming using a supercritical fluid were carried out using poly(ε-caprolactone). It is a semi-crystalline, biodegradable polymer with a relatively low melting point. Additionally, it is characterized by its non-toxicity; high hydrophobicity, which prevents cells from adhering to the raw material; good miscibility and mechanical compatibility in a mixture with other polymers; and relatively slow degradation in the physiological environment.

The blowing agent for the polymer material was carbon dioxide with a purity of 4.5 (over 99.995 vol.%), from Linde Gaz Polska (Warsaw, Poland).

The main part of the research used composite materials made of poly(ε-caprolactone) enriched with solid particles of porogens. In Table 1, basic data characterizing the raw materials used in the production of the composite materials is summarized (the material characteristics were provided by the suppliers).

### 2.2. Procedures for Foaming Materials Using Supercritical Carbon Dioxide

For the foaming process, a polymer without admixtures of pore-forming substances and a composite material based on PCL were used, in the same way as described in a previous work [36]. In order to obtain composite materials from PCL, a suitable amount of polymer granulate was melted using a heating plate, to which the appropriate amounts of blowing agent particles were added in batches, whilst ensuring the continuous manual mixing of the resulting mixture. When a composite with uniformly distributed porogen particles (confirmed by SEM analysis) was obtained, it was allowed to cool, and then cut into small pieces. Using the above-described method, both single-component and multi-component composite materials with varying proportions of individual components were produced.

**Figure 1:**
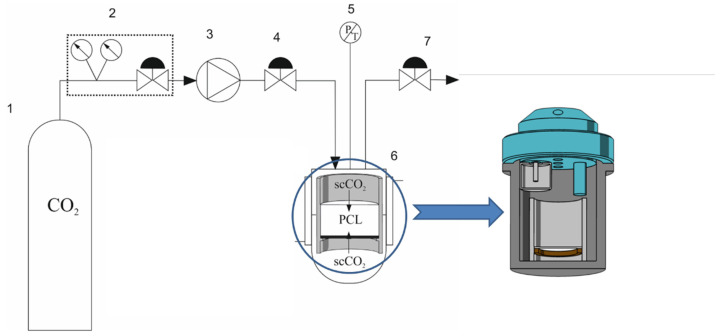
High-pressure test system diagram, where 1 is the carbon dioxide cylinder, 2 is the CO_2_ cylinder pressure reducer, 3 is the supercritical fluid pump, 4 is the valve, 5 is the pressure and temperature transducer, 6 is the thermostated high-pressure foaming chamber, and 7 is the back pressure regulator.

In order to carry out the foaming process, a high-pressure test system was designed, constructed and launched, the diagram of which is shown in Figure 1.

**Figure 2:**
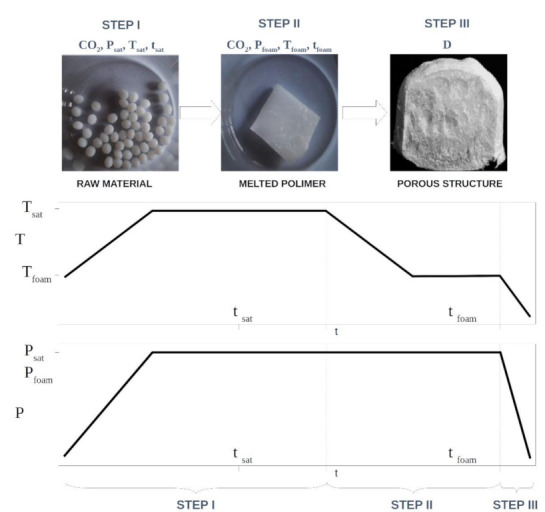
Procedure for the process of foaming polymer materials using supercritical carbon dioxide, as well as the variability of the process conditions.

The foaming of the polymer material using supercritical carbon dioxide was carried out in a batch process. The diagram showing the three-step process of producing solid foams is presented in Figure 2.

In the first step of the solid-foam production process, approximately 3 g of the previously prepared raw material was weighed and placed in a specially designed stainless-steel container (Figure 1) equipped with a perforated bottom coated with Teflon, allowing liquid diffusion from above as well as from below the polymer material. Then, the tank was placed in a high-pressure chamber (6) with a 100 cm^3^ reactor (100 mL High Pressure Autoclave, Amar Equipment, Mumbai, India). The expanded material in the sealed reactor was initially melted and saturated with carbon dioxide in the appropriate conditions. To this end, the gas bottle (1) was unscrewed and the heating mantle was turned on in order to set the appropriate saturation temperature T_sat_. After setting the temperature, the pump (SFT-10 piston pump, Supercritical Fluid Technologies, Newark, Delaware, USA) was used to dispense CO_2_ in the supercritical state (3), and the gas flow rate was set. Using the back pressure regulator (7), the saturation pressure P_sat_ in the thermostated high-pressure chamber (6) was set. When the correct values of the T_sat_ and P_sat_ operating parameters were reached, the carbon dioxide supply was stopped, and the valve (4) supplying gas to the reactor was closed. From this moment, the key stage of the first step of the foaming process began, which consisted in melting and saturating the composite material with supercritical carbon dioxide at pressure P_sat_ and temperature T_sat_ for a specified time t_sat_. The diffusion of carbon dioxide into the polymer material led to the plasticization of the material and the reduction of its glass transition temperature. Gas absorption led to the formation of a homogeneous polymer/CO_2_ material mixture. Then, the mixture obtained in this way was subjected to subsequent foaming steps II and III. The test system was subjected to cooling to T_foam_, while maintaining a constant pressure value P_foam_, which initiated the process of the nucleation of gas bubbles inside the polymeric material. For this purpose, the heating of the heating jacket of the high-pressure chamber (6) was turned off, the valve (4) supplying gas to the reactor was opened, and the pump (3) was turned on. After reaching the desired temperature value T_foam_, the system was left in the same conditions for the appropriate time t_foam_. The last important point of conducting the foaming process (step III) was the decompression of the system with the appropriate decompression rate D using a reducing valve. The pressure reduction shifted the thermodynamic equilibrium of the mixture and achieved gas supersaturation, which led to an increase in the pores in the solid-foam structure. After removing the sample from the high-pressure chamber, it was weighed and then left in ambient conditions for 24 h in order to remove residual carbon dioxide from the porous structure of the solid foam produced. In Figure 2, a general scheme of the investigated foaming process, including the variability of the process parameters (temperature and pressure) over time, is presented; in Table 2, the details of the variability of the parameters for the implementation of the three-step process of producing solid foams from poly(ε-caprolactone) and polymer composite materials are summarized.

### 2.3. Characterization of the Obtained Porous Structures

A key part of the experimental study was the analysis of the properties of the obtained porous structures using specialized analytical methods. In order to perform a quantitative and qualitative analysis enabling the presentation of relevant parameters in the context of applications in biomedical engineering, various analytical methods—scanning electron microscopy (SEM), computer microtomography (µ-CT), differential scanning calorimetry (DSC), and a static compression test—were applied.

The porosity of the obtained porous structures was determined using the gravimetric method. The sample was weighed 24 h after its removal from the high-pressure chamber, while its porosity was calculated by taking into account the approximation of the sample shape to a cylinder of height h [cm] and diameter d [cm]. Then, the density of the solid foam was calculated according to the following relationship:

(1) $$\rho_{\mathrm{foam}}=\frac{m_{\mathrm{foam}24}}{V_{\mathrm{foam}}}$$

where ρ_foam_ is the solid foam density (g/cm^3^), m_foam24_ is the sample mass 24 h after its removal from the reactor (g), and V_foam_ is the sample volume (cm^3^).

The porosity ε of the sample was determined using the following relationship:

(2) $$\varepsilon =\left(1-\frac{\rho_{\mathrm{foam}}}{\rho_{\mathrm{PCL}}}\right)\cdot 100\%$$

The density of poly(ε-caprolactone) at room temperature is ρ_PCL_ = 1.145 g/cm^3^.

The qualitative analysis of the solid foams was performed using photographic images, a scanning electron microscope (SEM) and a computer microtomograph. For this purpose, specialized computer programs for image analysis were used.

The analysis of the morphology of the lateral surface and cross-section, after spraying with a gold conductor layer (sputtering machine EMITECH K550X by Quantum Technologies, Tychy, Poland), was performed using the SEM Phenom World PRO (ThermoFisher Scientific, Warsaw, Poland) scanning electron microscope at two different magnifications (600×, 2000×). In order to determine the basic values describing the morphology of porous structures, the surface area of 100 different pores visible in the SEM photos was determined for each of the obtained samples. The characteristic dimension for the determination of the pore size was the equivalent diameter corresponding to a circle with the same area; the pore diameter was calculated according to the following formula:

(3) $$d=\sqrt{\frac{4\cdot P}{\text{π}}}\;$$

where d is the diameter of one of the analyzed pores [μm], and P is the surface area of one of the analyzed pores [μm^2^].

Based on the obtained numerical values for 100 pore diameters, the following parameters were determined: the minimum diameter (d_min_), the maximum diameter (d_max_), and the average numerical pore size (d_10_). Then, using the obtained data, the average pore size weighted by the volume d_43_ and the average pore density per volume unit (N) were calculated.

In order to determine the pore-size distribution, the diameters were divided into 12 classes with a width of 25 µm each. The size of each of the pores was allocated to only one of the classes, and the numerical fractions representing each of them were calculated. The pore-size distribution was determined by comparing the numerical fractions of all of the classes on the graph.

Computed microtomography (µCT) made it possible to map the internal structure of the tested object on the basis of two-dimensional projections recorded at different angles. The ΜCT images were taken on a SkyScan 1172 computer microtomograph from BRUKER (Warsaw, Poland). When taking a measurement, the sample rotates by an angle of 360°, stopping at certain angular steps, while the radiation source and detector remain motionless. At each of the achieved angular positions, a transmission image (projection) of the image is taken.

The static compression test was carried out using the Instron 5566 (Opole, Poland) testing machine. In order to carry out the strength analysis, each of the produced solid foams was cut into 5 cuboidal samples with dimensions of ~ 5 mm × 5 mm × 8 mm. For the experiment, constant values of the following parameters were set: the speed of the lowering of the measuring beam was 0.4 mm/min, and the deformation of the test at the maximum compressive stress was 70%. On the basis of the analysis, the Young’s modulus and the maximum strength of the solid foam were determined.

The thermal analysis was performed by the Differential Scanning Calorimetry (DSC) method using a multi-module TGA/DSC/FTIR/QMS device from METTLER TOLEDO. (Warsaw, Poland) This device has a built-in thermobalance with a very high sensitivity, and it allows one to study phase changes in various atmospheres from room temperature to 1600 °C. The measuring principle in the TGA/DSC thermal analyzer is based on the measurement of the heat flow difference between the test sample and the reference sample (an empty crucible) analyzed under the same conditions. The thermal analysis was performed in an inert gas atmosphere of argon, the flow of which was 30 mL/min. A sample of the tested material was heated in the temperature range of 30–120 °C, and the heating rate was 10 °C/min. Based on the results, the degree of crystallinity and the melting point of the material were determined.

## 3. Results and Discussion

### 3.1. Influence of the Foaming Parameters on the Morphology and Mechanical Properties of Polymer Foams

**Figure 3:**
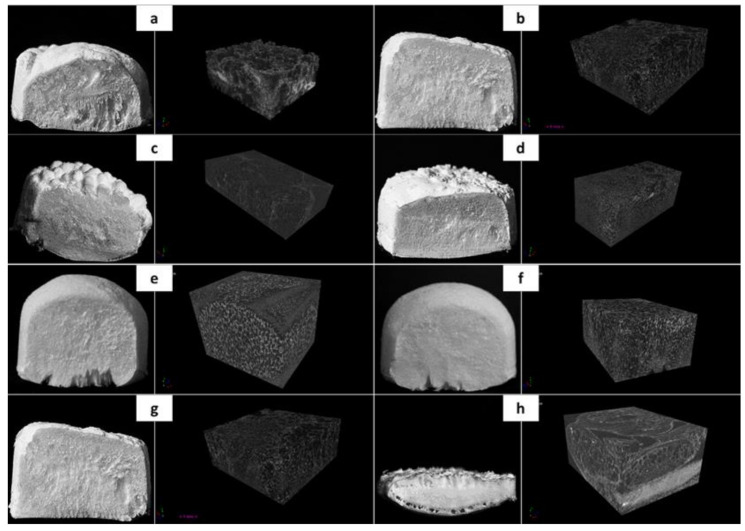
Qualitative analysis of solid foams obtained as a result of poly(ε-caprolactone) foaming in the following process conditions: (**a**) T_sat_ = 70 °C, P_sat_ = 9 MPa, t_sat_ = 1 h; (**b**) T_sat_ = 70 °C, P_sat_ = 18 MPa, t_sat_ = 1 h; (**c**) T_sat_ = 50 °C, P_sat_ = 18 MPa, and t_sat_ = 1 h; (**d**) T_sat_ = 100 °C, P_sat_ = 18 MPa, and t_sat_ = 1 h; (**e**) T_sat_ = 70 °C, P_sat_ = 18 MPa, and t_sat_ = 0.5 h; (**f**) T_sat_ = 70 °C, P_sat_ = 18 MPa, and t_sat_ = 6 h; (**g**) T_sat_ = 70 °C, P_sat_ = 18 MPa, t_sat_ = 1 h, and D_fast_; and (**h**) T_sat_ = 70 °C, P_sat_ = 18 MPa, t_sat_ = 1 h, and D_slow_. The presented photos come from a camera and a computer microtomograph.

In order to select the appropriate conditions to carry out the composite material foaming process, polymer foaming experiments were performed. A quality analysis was performed on the basis of photographic images and a computer microtomograph (see Figure 3). Based on the combined photos, a significant influence of the parameters of the foaming process on the morphology properties of solid foams was demonstrated. The increase in pressure, temperature and saturation time leads to the obtaining of structures characterized by a greater homogeneity of morphology. In addition, the decompression rate of the system plays a key role in the quality of the products produced. The use of rapid expansion allows us to obtain the effect of the uniform foaming of the polymeric material throughout its volume.

An analysis related to the effect of the concentration and conditions of the foaming process on the properties of the solid foams was carried out.

#### 3.1.1. Nanohydroxyapatite

**Figure 4:**
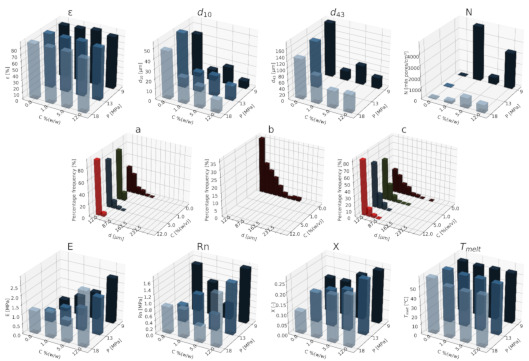
The effects of the concentration of the nHA additive used and the pressure (9–18 MPa) for a constant temperature (70 °C) and saturation time (1 h) on the parameters describing the morphology of PCL solid foams: porosity (ε), average pore size (d_10_), mean pore size weighted by volume (d_43_), mean pore volume density (N), and pore-diameter distribution for pressures of (**a**) 9 MPa, (**b**) 13 MPa, and (**c**) 18 MPa, for the mechanical properties of the Young’s modulus (E) and mechanical strength (Rn), and the thermal analysis of the degree of crystallinity (X) and melting point (T_melt_).

**Figure 5:**
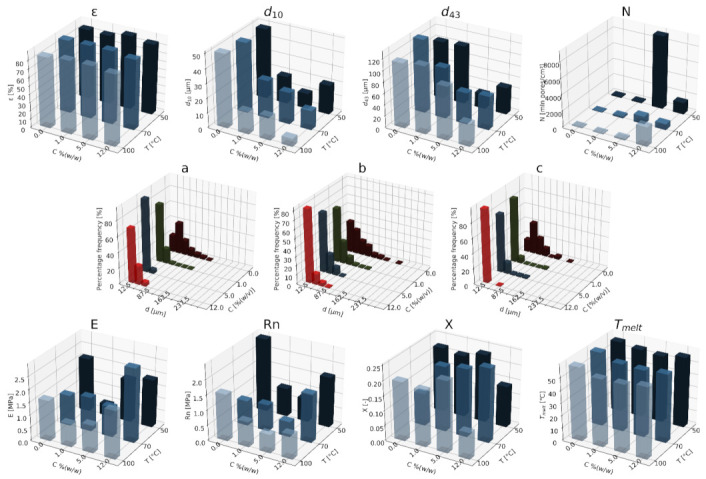
Effects of the concentration of the applied additive nHA and the temperature (50–100 °C) for a constant pressure (18 MPa) and saturation time (1 h) on the parameters describing the morphology of solid PCL foams: porosity (ε), average pore size (d_10_), volume weighted average pore size (d_43_), average pore volume density (N), pore-diameter distribution for temperatures of (**a**) 50 °C, (**b**) 70 °C, and (**c**) 100 °C, for the mechanical properties of the Young’s modulus (E) and mechanical strength (Rn), and for the thermal analysis of the degree of crystallinity (X) and melting point (T_melt_).

**Figure 6:**
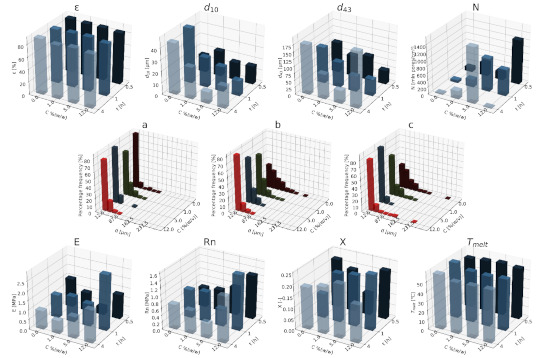
The effect of the concentration of the nHA additive used and the time (0.5–4 h) for a constant temperature (70 °C) and saturation pressure (18 MPa) on the parameters describing the morphology of PCL solid foams: porosity (ε), average pore size (d_10_), volume-weighted average pore size (d_43_), average pore volume density (N), and pore-diameter distribution for times of (**a**) 0.5 h, (**b**) 1 h, (**c**) 4 h, for the mechanical properties of the Young’s modulus (E) and mechanical strength (Rn), and for the thermal analysis of the degree of crystallinity (X) and melting point (T_melt_).

In Figure 4, Figure 5 and Figure 6, a summary of the variability of the parameters of the morphology and mechanical properties of porous structures obtained by the foaming of the composite material (PCL/nHA) for four concentrations of the additive used (0–12% (*w/w*) nHA) for variable pressures (9–18 MPa), temperatures (50–100°C) and saturation times (0.5–4 h) are presented.

The obtained porous structures are characterized by a porosity exceeding 80%. The highest values were obtained for solid foams produced under a saturation pressure of 18 MPa. In Figure 4, no significant changes in the value of the porosity of the solid foams were observed as a result of the applied porogen concentrations under the conditions of variable saturation temperatures. Figure 6 shows a slight decrease in this parameter due to the increase in the porogen content. The most appropriate process conditions are 1 h of saturation.

The use of a blowing agent leads to a decrease in the value of the average pore size compared to structures made of pure PCL. On the basis of the obtained relations, it seems that 18 MPa is the most suitable saturation pressure to obtain structures with the desired properties. The addition of another portion of nHA reduces the value of the pore diameter d_43_. Under a relatively high saturation pressure, the observed dependencies seem to be more unambiguous. In all of the composite systems, structures with pores smaller than those made of pure polymer were obtained. The most desirable values of this parameter were obtained for the foam containing 5% (*w/w*) nHA produced at the saturation temperature of 70 °C. The pores density increases with an increasing porogen content and with and increasing saturation temperature. The structures with the highest number of pores were obtained at a saturation temperature of 100 °C. As the temperature increases, the dependence of the pore size on the additive concentration becomes more clear-cut—the diameter decreases as the amount of pore-forming agent used increases. This effect is related to the possibility of heterogeneous nucleation with the presence of additional unmelted polymer crystallites at a low temperature (50 °C). The increase in the concentration of the pore-forming substance leads to a decrease in the pore size of d_43_, with the lowest values of this parameter being obtained as a result of foaming at a low temperature (50 °C). When analyzing the list containing changes in the value of the mean pore diameter under the conditions of a variable porogen content and saturation time of the polymeric material, a decrease in the value of this parameter was observed as a result of the increase in the nHA concentration. The exception is the foams saturated for 4 h, where an initial decrease in the mean pore size was noticed with increasing nHA content, with 12% (*w/w*) nHA again increasing the value of this parameter.

The highest values of the maximum pore size are obtained when the material is saturated with gas for 4 h. For shorter times (0.5–1 h), the addition of the blowing agent leads to a decrease in the value of the pore size. For 4 h of saturation time, there was initially a decrease, and then for 12% (*w/w*) nHA a significant increase in the value of the maximum pore size was observed. An increase in the concentration of the blowing agent and a decrease in the saturation time leads to a decrease in the pore diameter d_43_.

As the saturation pressure increases, the pore-diameter distribution shifts to a smaller size. For composites with various nHA contents, the solid foams produced under the saturation pressure of 9 MPa are the most homogeneous. Moreover, it was observed that solid foams containing 12% (*w/w*) nHA showed the greatest homogeneity of the structure in comparison to the others.

In all of the variants of the process conditions, the addition of porogen leads to a narrowing of the pore diameters’ range, and to obtaining solid foams with a more homogeneous structure. As the impregnation temperature increases, structures of greater homogeneity are obtained. As the saturation time increases, the pore-size distribution shifts towards larger diameters. Within the whole range of parameters considered, the most homogeneous structures were obtained in the conditions of a saturation time equal to 0.5 h, and for the composite material containing 12% (*w/w*) of nHA pore-forming particles.

The above Figure 4, Figure 5 and Figure 6 present the characteristics of the produced solid foams in terms of their mechanical properties and crystallinity. As the nHA content increased, an increase in the value of the Young’s modulus was observed. In most of the presented cases, a higher value of this parameter was demonstrated for solid foams obtained as a result of foaming under the pressure of 18 MPa. Generally, each time a portion of the porogen is added, the mechanical strength increases. In the case under consideration, the exception was a composite containing 5% (*w/w*) nHA. The addition of successive portions of porogen leads to an increase in the Young’s modulus, the mechanical strength and the degree of crystallinity of the porous structure (Figure 6). Moreover, a slight decrease in the value of this parameter was observed for composite materials in relation to the pure polymer. On the basis of the combined results, with the increase of the nHA concentration, solid foams with increasing values of Young’s modulus, mechanical strength and the degree of crystallinity are obtained. It seems that 1 h is the most appropriate gas saturation time for the polymeric material to obtain structures with appropriate properties.

In the studied range of pressure variability and pore-forming substance concentration, an increase in the crystalline nature of solid foams was observed with the increase in nHA concentration. On the other hand, the melting point value remains at a relatively constant level over the entire range of the parameter variability, and amounts to nearly 60 °C.

#### 3.1.2. Nanocellulose

**Figure 7:**
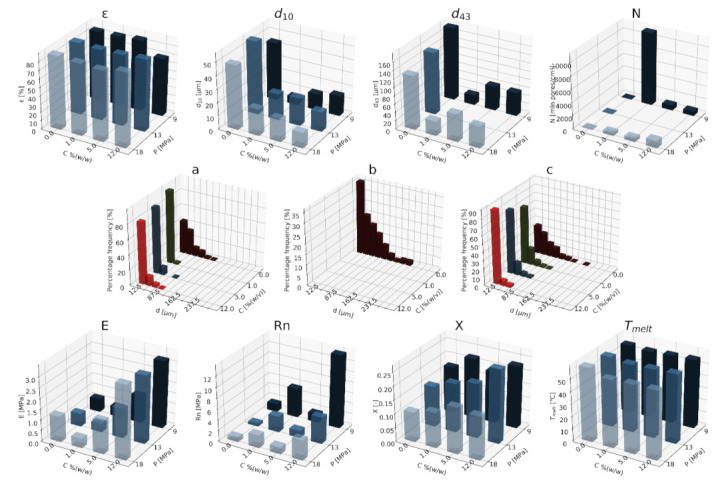
The effect of the concentration of the nC additive used and the pressure (9–18 MPa) for a constant temperature (70 °C) and saturation time (1 h) on the parameters describing the morphology of PCL solid foams: porosity (ε), average pore size (d_10_), mean pore size weighted by volume (d_43_), mean pore volume density (N), and pore-diameter distribution for pressures of (**a**) 9 MPa, (**b**) 13 MPa, (**c**) 18 MPa, for the mechanical properties of the Young’s modulus (E) and mechanical strength (Rn), and for the thermal analysis of the degree of crystallinity (X) and melting point (T_melt_).

**Figure 8:**
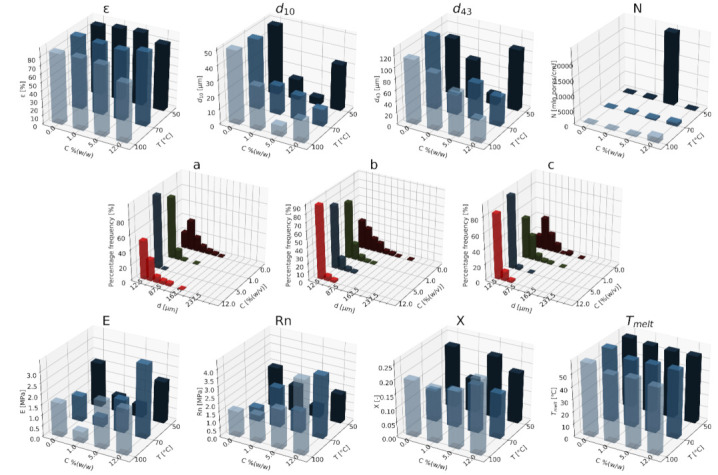
Effect of the concentration of the applied additive nC and the temperature (50–100 °C) for a constant pressure (18 MPa) and saturation time (1 h) on the parameters describing the morphology of solid PCL foams: porosity (ε), average pore size (d_10_), volume weighted average pore size (d_43_), average pore volume density (N), and pore-diameter distribution for temperatures of (**a**) 50 °C, (**b**) 70 °C, and (**c**) 100 °C, for the mechanical properties of the Young’s modulus (E) and mechanical strength (Rn), and for the thermal analysis of the degree of crystallinity (X) and melting point (T_melt_).

**Figure 9:**
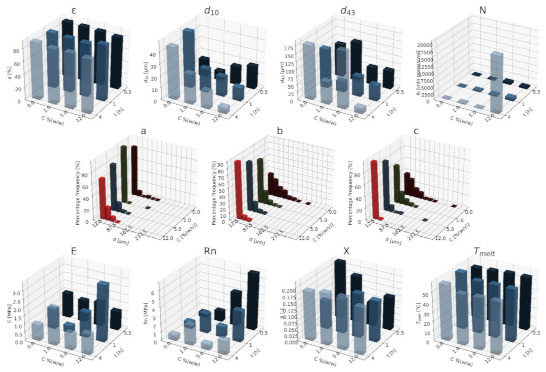
The effect of the concentration of the nC additive used and the time (0.5–4 h) for a constant temperature (70 °C) and saturation pressure (18 MPa) on the parameters describing the morphology of PCL solid foams: porosity (ε), average pore size (d_10_), volume-weighted average pore size (d_43_), average pore volume density (N), and pore-diameter distribution for times of (**a**) 0.5 h, (**b**) 1 h, (**c**) 4 h, for the mechanical properties of the Young’s modulus (E) and mechanical strength (Rn), and for the thermal analysis of the degree of crystallinity (X) and melting point (Tmelt).

In Figure 7, Figure 8 and Figure 9, a summary of the variability of the morphological and strength parameters of porous composite (PCL/nC) structures is presented.

The obtained solid foams are characterized by a porosity of over 80%. With an increase in the nC content, the porosity slightly increases when a relatively high saturation pressure is used (13, 18 MPa). Moreover, the addition of an nC portion leads to a drastic decrease in the mean pore size d_10_ compared to pure PCL. The increase in concentration for the two pressures used (13 and 18 MPa) reduces the pore diameter, while under the saturation pressure of 9 MPa, the opposite effect was identified. The increase in pressure increases the mean pore size. The addition of a blowing agent leads to a drastic decrease in the value of d_43_. Moreover, with increasing pressure, diameter d_43_ also decreases. For the application of saturation temperatures of 50 and 100 °C, the dependencies of the Young’s modulus and mechanical strength on the concentration of nC are very similar. Additionally, as before, an increase in the concentration of the pore-forming substance and the temperature leads to a decrease in the value of pore size d_43_. For saturations lasting 0.5 h, a slight decrease in the mean pore diameters was observed as a result of the addition of the blowing agent unit compared to the higher saturation times. Moreover, as a consequence of using this saturation time, an increase in the diameter values for the increasing concentrations was identified. For the remaining saturation times (1–4 h), the increase in concentration causes a clear decrease in the pore size.

More homogeneous structures were obtained in conditions with a relatively low saturation pressure (9 MPa). For 18 MPa, with an increase in the nC concentration, the pore-size distribution shifts towards the lower dimensions, while for 9 MPa, the concentration of 1% (*w/w*) nC is an exception. By applying a pressure of 9 MPa, structures with a relatively high homogeneity of small pores distribution were obtained. As the temperature increases, the homogeneity of the structure decreases. In the high temperature range (70 and 100 °C), along with an increase in the concentration of the additive, the distribution shifts towards smaller pores. At 50 °C the general relationship is also maintained, with the exception to this rule being the addition of 12% (*w/w*) nC. The use of a relatively low saturation time (0.5 h) leads to structures of the highest homogeneity. The effect of the additive concentration for specific impregnation conditions on the properties of solid foams is ambiguous. In general, as before, the use of larger amounts of the additive shifts the distribution towards smaller pore sizes. For pure PCL, as the saturation time increases, the distribution shifts towards the larger pores, but the homogeneity of the porous structure also decreases.

The value of the Young’s modulus of solid foams increases with the increase of the applied concentration of nC. In the tested range of variability, an increase in mechanical strength was demonstrated as a result of increasing the content of the pore-forming substance, and for each 5% (*w/w*) nC, the occurrence of a characteristic minimum value of this parameter was identified. The most rigid structures were obtained in conditions of a relatively low saturation pressure (9 MPa), while a high pressure (18 MPa) causes the formation of structures characterized by high elasticity. As the applied concentration of nC increased at variable saturation temperatures, an increase in the values of the mechanical parameters was observed regarding the Young’s modulus and mechanical strength. In the range of low concentrations (0–1% (*w/w*) nC), the most elastic structures were obtained at a saturation temperature of 50°C, and in the range of high concentrations (5–12% (*w/w*) nC), as the temperature increased (50–100 °C), solid foams were obtained with higher mechanical strength. In general, in the tested range of parameters of the porogen concentration and saturation time, an increase in the value of the Young’s modulus, mechanical strength and degree of crystallinity was observed. In the high concentration range (5–12% (*w/w*) nC), solid foams saturated for 1 h during production showed greater elasticity than those saturated for 0.5 or 4 h. For low concentrations (0–1% (*w/w*) nC), as the saturation time increased (0.5–4 h), more rigid structures with greater mechanical strength were obtained. In the range of higher concentrations (5–12% (*w/w*) nC), the opposite effect was obtained; for the saturation time equal to 0.5 h, structures of superior mechanical strength were obtained.

As both the concentration of the additive and the saturation pressure increased, a slight increase in the degree of crystallinity of the solid foams was observed. Within the tested range of variability of conditions, the melting point of the foams was close to 60 °C. As the concentration of nC increased, a slight increase in the melting point was observed. More crystalline structures were obtained for increasing contents of nC at a variable saturation temperatures. The lowest value of the degree of crystallinity was obtained for the foams produced at 70 °C. The melting point of the scaffolds produced is below 60 °C. For the saturation times of 1 and 4 h, more crystalline structures were obtained as the concentration increased. Increasing the concentration of 5–12% (*w/w*) nC does not cause significant changes in the value of this parameter. By saturating the material for 0.5 h, a decrease in the degree of crystallinity was observed as the concentration of the additive used increased.

#### 3.1.3. Nanographene Oxide

**Figure 10:**
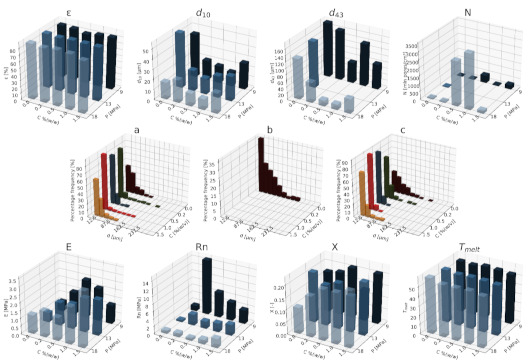
The effect of the concentration of the nGO additive used and the pressure (9–18 MPa) for a constant temperature (70 °C) and saturation time (1 h) on the parameters describing the morphology of PCL solid foams: porosity (ε), average pore size (d_10_), mean pore size weighted by volume (d_43_), mean pore volume density (N), and pore-diameter distribution for pressures of (**a**) 9 MPa, (**b**) 13 MPa, (**c**) 18 MPa, for the mechanical properties of the Young’s modulus (E) and mechanical strength (Rn), for the thermal analysis of the degree of crystallinity (X) and melting point (T_melt_).

**Figure 11:**
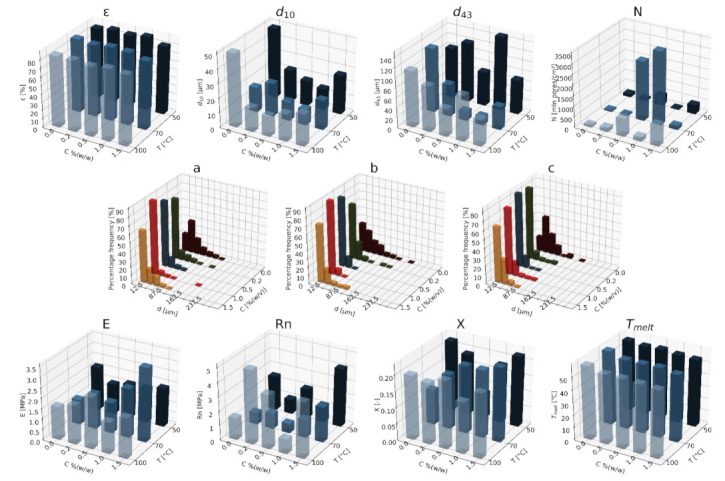
Effect of the concentration of the applied additive nGO and the temperature (50–100 °C) for a constant pressure (18 MPa) and saturation time (1 h) on the parameters describing the morphology of solid PCL foams: porosity (ε), average pore size (d_10_), volume-weighted average pore size (d_43_), average pore volume density (N), and pore-diameter distribution for temperatures of (**a**) 50 °C, (**b**) 70 °C, and (**c**) 100 °C, for the mechanical properties of the Young’s modulus (E) and mechanical strength (Rn), and for the thermal analysis of the degree of crystallinity (X) and melting point (Tmelt).

**Figure 12:**
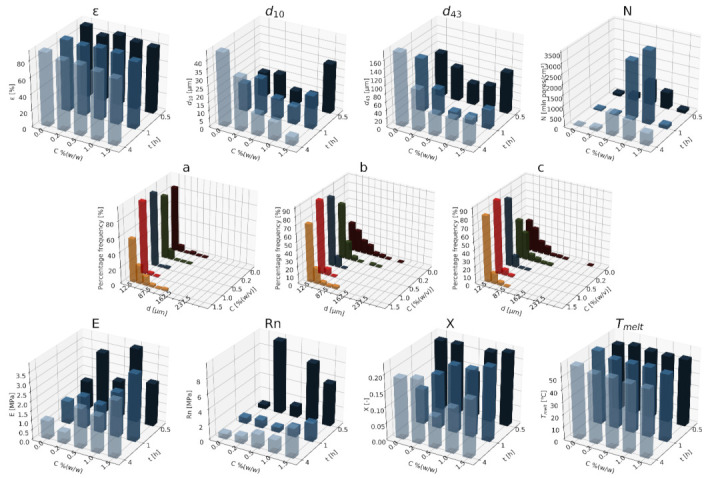
The effect of the concentration of the nGO additive used and the time (0.5–4 h) for a constant temperature (70 °C) and saturation pressure (18 MPa) on the parameters describing the morphology of PCL solid foams: porosity (ε), average pore size (d_10_), volume-weighted average pore size (d_43_), average pore volume density (N), and pore-diameter distribution for times of (**a**) 0.5 h, (**b**) 1 h, and (**c**) 4 h, for the mechanical properties of the Young’s modulus (E) and mechanical strength (Rn), and for the thermal analysis of the degree of crystallinity (X) and melting point (Tmelt).

In Figure 10, Figure 11 and Figure 12, the variability of the morphological and strength parameters of porous structures obtained by foaming the composite material (PCL/nGO) for five concentrations of the nGO additive used (0–1.5% (*w/w*)) for variable pressures in the range of 9–18 MPa, temperatures of 50–100 °C, and saturation times of 0.5–4 h is presented.

The obtained solid foams are characterized by a porosity close to 80%. In the range of low concentrations (0–1% (*w/w*) nGO) under a relatively high pressure (18 MPa), foams showing a higher porosity value than those in the low pressure range (9–13 MPa) were obtained. In the whole range of concentrations, the solid foams saturated for 1 h showed the highest value of this parameter. As the concentration of the additive increased, a decrease in the porosity value was observed.

The addition of a porogen leads to a sharp decrease in the pore size of d_10_; then, as the concentration increases, this parameter increases slightly. The value of the d_43_ diameter decreases with an increasing nGO concentration and saturation pressure. Under the conditions of 50 and 70 °C saturation temperatures in the range of low concentrations (0.2–1.0% (*w/w*) nGO), there was a decrease in the mean pore size and an increase in the mean pore surface density as the porogen content increased, while for the concentration of 1.5% (*w/w*) nGO, the opposite effect was observed. At a temperature of 100 °C, a decrease in the mean pore size in the concentration range of 0–0.5% (*w/w*) nGO was identified, and it increased again for the use of higher concentrations (0.5–1.5% (*w/w*) nGO). On the basis of the above data, it can be concluded that the addition of 1.0% (*w/w*) nGO is a critical point for supersaturation. The addition of a blowing agent leads to a reduction in the pore diameter d_43_. As the concentration of nGO increased under the conditions of variable saturation times, the mean pore size decreased, while for a given concentration, an increase in this parameter was observed with the extension of the saturation time. Composite foams produced with the addition of a blowing agent of 1.5% (*w/w*) nGO are an exception to the indicated dependence. In the range of low concentrations (0–1% (*w/w*) nGO), the foams foamed for a short time (0.5 h) contained a larger number of pores compared to the structures saturated for a longer time. Based on the presented data, it has been shown that the use of 1 h of impregnation of the composite material with a graphene oxide content of 0.2% (*w/w*) nGO allows us to obtain foams with a maximum pore size exceeding 150 µm.

As the concentration of the used additive increased, structures characterized by greater homogeneity were obtained; in addition, the distribution of the pore diameters was shifted towards smaller sizes. The structures obtained under low pressure were more homogeneous than those obtained under high pressure. Under high pressure (18 MPa), structures containing more and smaller pores are obtained. As the concentration increases, the pore-size distribution shifts towards smaller diameters. The addition of a pore-forming substance leads to an increase in the homogeneity of the porous structures. As the saturation time increases, the pore-diameter distribution shifts to larger sizes, and increasing the nGO concentration leads to a reduction in the pore-size range. The most homogeneous structures were obtained in the range of higher concentrations of 0.5–1.5% (*w/w*) nGO, and at a relatively short saturation time (0.5 h).

In the range of high pressures of 13–18 MPa, as the concentration increased, an increase in the elasticity of the solid foams was observed, while for the pressure of 9 MPa, an increase in the value of this parameter was identified for concentrations of 0–0.5% (*w/w*) nGO. Then, for 1% (*w/w*) nGO, a flattening of the relationship was observed; finally, for 1.5% (*w/w*) nGO, a decrease in the value of Young’s modulus was observed. Under high pressure conditions of 13–18 MPa, the increase in concentration leads to the obtaining of structures with greater strength, while for 9 MPa the opposite effect was observed. As the concentration increased, an increase in the mechanical parameters of the solid foams was observed. The most significant effect of the concentration of the applied additive on the value of Young’s modulus was observed in the case of foams produced at the saturation temperature of 70 °C. For 100 °C in the range of low concentrations of the blowing agent (0–0.5% (*w/w*) nGO), an increase in the Young’s modulus was identified, while high concentrations of 0.5–1.5% (*w/w*) nGO lead to a decrease in the elasticity of the foam. Analyzing the dependence of the strength on the concentration of the additive used, an increase in the value of this parameter was noticed along with the increase in the content of nGO. Under the conditions of a saturation temperature equal to 70 °C in the tested concentration range, the obtained structures were characterized by a higher mechanical strength than those at 50 and 100 °C. Generally, for the saturation temperature of 1 and 4 h, the increase in the concentration of nGO leads to an increase in the values of the mechanical parameters. The structures that are saturated for 0.5 h and have a relatively low content of graphene oxide of 0.2% (*w/w*) are characterized by the highest mechanical strength.

In the range of high concentrations of the pore-forming substance (0.5–1.5% (*w/w*) nGO) at a pressure of 18 MPa, structures characterized by a relatively high degree of crystallinity were obtained. The melting point of the obtained solid foams was below 60 °C, and remained at a constant level in the tested range of conditions’ variability. Generally, in the concentration range studied, foams impregnated at 50 and 70 °C showed an increased degree of crystallinity as the nGO concentration increased. The foams saturated at 100 °C are a special case, as an increase in the degree of crystallinity was observed in the concentration range of 0–0.5% (*w/w*) nGO, then for concentrations of 0.5–1% (*w/w*) there was a slight decrease in the value of this parameter, and for the concentration range of 1–1.5% (*w/w*) nGO again increased. The melting point in the tested range was below 60 °C. The melting point value for the foams saturated for 1 and 4 h was slightly higher than that obtained for the shorter saturation time of 0.5 h.

### 3.2. Influence of the Type of Porogen Particles on the Favorable Properties of Composite Structures

In order to analyze the impact of the nature of the applied pore-forming substance additives, the results of the foaming process of the polymer material enriched with nanometric particles of the following porogens with different contents were compiled for nanohydroxyapatite (nHA), nanocellulose (nC) and graphene nanoxide (nGO), with conditions of pressure (18 MPa), temperature (70 °C), saturation time (1 h) and immediate decompression rate.

**Figure 13:**
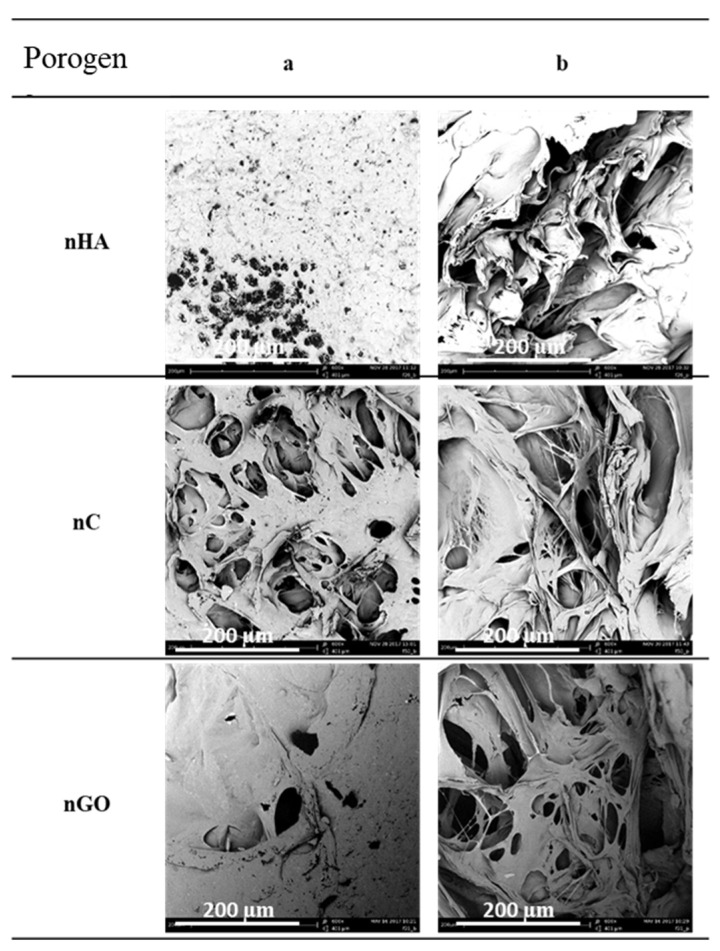
Qualitative analysis of the solid foams obtained as a result of foaming a composite material containing 1% (*w/w*) of one-component additive nHA, nC, or nGO under constant pressure (18 MPa) for a constant temperature (70 °C) and time (1 h) of impregnation: (**a**) side surface and (**b**) cross-section.

A qualitative analysis was carried out on the basis of the SEM scanning electron microscope photos of the lateral surface and the cross-section (see Figure 13). All of the obtained solid foams showed a porosity exceeding 80%. Solid foams containing a low content of graphene oxide are characterized by a relatively high surface pore density. The average pore size in the studied range of porogen variability is within a relatively constant range of values, not exceeding 30 µm. The highest values of the maximum pore size were obtained for the structures made of composite material containing 0.2% (*w/w*) graphene oxide. In each of the cases under consideration, the pore diameter d_43_ decreases in relation to foams produced without porogens due to the addition of a blowing agent.

**Figure 14:**
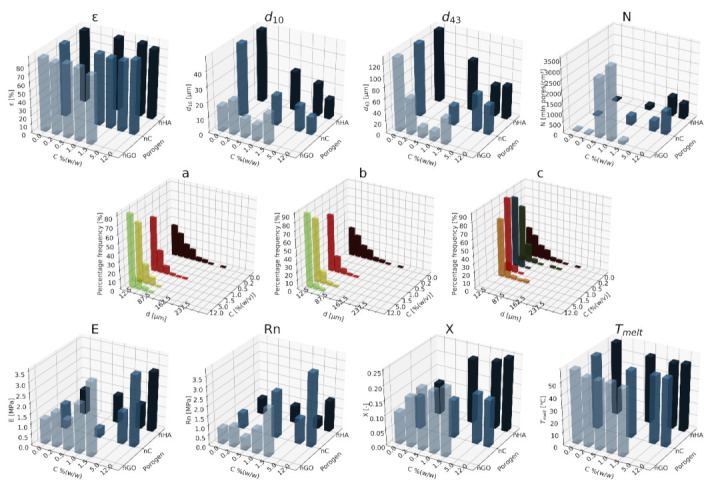
Analysis of the properties of solid foams taking into account the type of porogens, where ε is the porosity, d_10_ is the average pore diameter, d_43_ is the volume-weighted pore diameter, N is the pore-volume density, Rn is the mechanical strength, E is the Young’s modulus, X is the degree of crystallinity, T_melt_- melting point, for homogeneities of (**a**) nHA, (**b**) nC, and (**c**) nGO.

In Figure 14, the effect of the type of the additive used on the homogeneity of the obtained porous structures is shown. For this purpose, data analysis was performed for 1% (*w/w*) of the used porogen nHA, nC, and nGO. Comparing the results of the use of the porogens to the polymer at the same concentration of 1% (*w/w*), the narrowest pore size range is characteristic for the foam made of a composite material containing 1% (*w/w*) of graphene oxide. The least homogeneous solid foams and the foams containing the largest range of pore diameters were obtained from polymeric materials enriched with nanometric hydroxyapatite particles. The most mechanically resistant structures and those characterized by a high degree of crystallinity are solid foams made of a polymer material enriched with hydroxyapatite and graphene oxide.

### 3.3. Influence of the Size of the Porogen Particles on the Favorable Properties of Composite Structures

In order to assess the impact of the particle size of the applied pore-forming substance additives, the results of the foaming process of the polymer material enriched with hydroxyapatite particles with two powder grain diameters—200 nm and 10 μm—were compiled, while the foaming was carried out in constant conditions of pressure (18 MP), temperature (70 °C), saturation time (1 h) and the immediate decompression rate.

**Figure 15:**
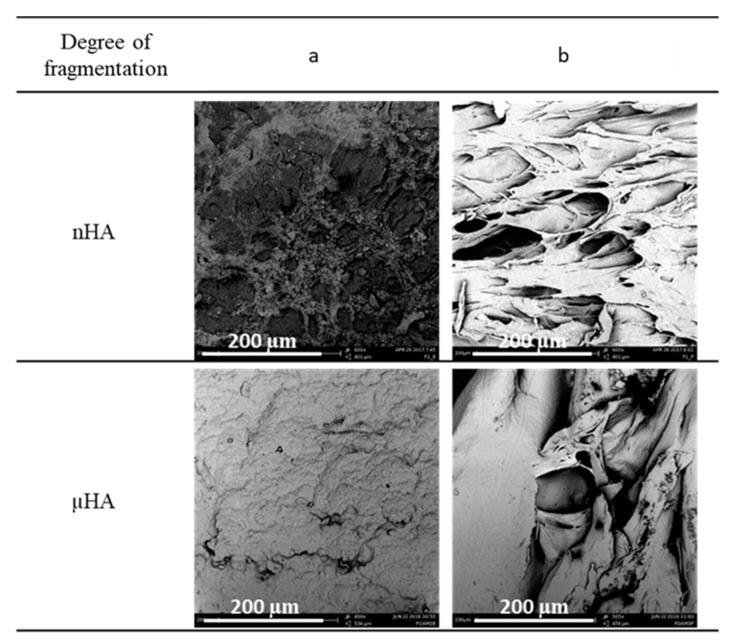
Qualitative analysis of solid foams obtained as a result of the foaming of a composite material containing hydroxyapatite in two degrees of fragmentation (nHA and µHA) at a constant pressure (18 MPa) for a constant temperature value (70 °C) and saturation time (1 h): (**a**) lateral surface and (**b**) cross-section.

In Figure 15, SEM photographs reveal the significant differences in both the size and shape of the pores of the obtained solid foams. The addition of nanoparticles leads to structures characterized by a large number of small pores with a slightly elongated shape, while the use of hydroxyapatite with a micro-particle size leads to the production of foams with large, round pores.

Figure 15 presents photos of the side surface and the cross-section of the obtained solid foams.

**Figure 16:**
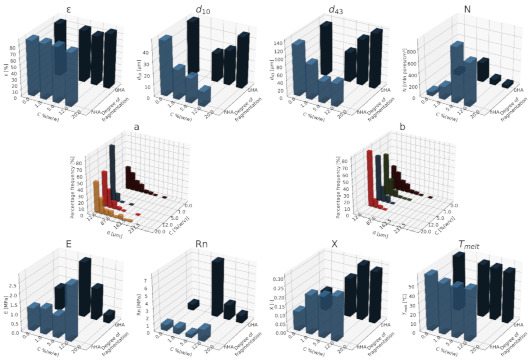
Analysis of the properties of solid foams, taking into account the degree of fragmentation of hydroxyapatite, where ε is the porosity, d_10_ is the average pore diameter, d_43_ is the volume-weighted pore diameter, N is the pore-volume density, Rn is the mechanical strength, E is the Young’s modulus, X is the degree of crystallinity, and T_melt_ is the melting point, for homogeneities of (**a**) µHA and (**b**) nHA.

The porosity values for both of the analyzed materials are in the range close to 80%. Based on the presented data, it has been shown that the structures made with the use of hydroxyapatite with larger particle sizes are characterized by a slightly larger mean pore size. On the basis of the collected data, it was shown that structures made with the use of hydroxyapatite with larger grain diameters are characterized by larger maximum pore diameters (over 100 µm) compared to structures enriched with nanometric particles of this porogen. On the basis of the compiled values of d_43_, two opposite dependencies of this parameter on the concentration of hydroxyapatite in relation to the degree of fragmentation of the pore-forming substance were observed. In the case of the use of a porogen with a grain size in the range of micrometers, an increase in the value of the d_43_ diameter was observed as the concentration of the additive increased; meanwhile, with the use of hydroxyapatite nanoparticles, the addition of another portion leads to a decrease in the pore size. In Figure 16, the effect of the particle size on the homogeneity of porous structures is shown. As the concentration of the porogen increases, the uniformity of the solid foams increases. The most homogeneous structures with a narrow range of pore-size distributions were obtained for structures made of polymeric materials enriched with nanometric hydroxyapatite particles.

The most durable and elastic structures were obtained for the 5% (*w/w*) addition of hydroxyapatite with a grain diameter in the micro scale, while the least durable structures were obtained for the application of 5% (*w/w*) nHA.

In the literature, many works are devoted to the process of foaming materials in order to obtain functional porous structures. Recently, there have been reports on the analysis of the process of the foaming of composite materials in order to obtain solid foams with appropriate properties. Chen’s team and co-authors publish papers on the foaming process with the use of two-stage decompression, which allows them to obtain porous structures with two pore sizes. The characteristics of the obtained structures allow them to be used in biomedical engineering for the regeneration of the bone weaver [33]. The next paper describes the method of foaming polystyrene, where foams were obtained which were characterized by a bimodal structure. In this work, as in ours, the possibility of controlling the properties of the obtained porous structures by adjusting the parameters of the manufacturing process was noticed [47]. In the next work, the PCL–CNC composite material was used for the production of porous structures by the extrusion method, where special attention was paid to the influence of the CNC concentration on the properties of the structures [48]. In that work, the results were similar to ours.

## 4. Conclusions

As a result of the research work, the following conclusions were drawn.

The properties of nHA/PCL, nC/PCL, nGO/PCL composites foamed with scCO_2_ were investigated and compared with those of pure PCL foams foamed under analogous conditions. It is possible to influence the properties of foams by changing the amount and type of porogen, and the foaming conditions. Significant effects of the operational parameters on the morphology and mechanical properties of solid foams were identified.

All of the solid foams are characterized by porosities exceeding 80%. A decrease in pressure and an increase in saturation time contributes to an increase in the diameter d_43_. Decreasing the pressure and saturation time leads to an increase in the uniformity of the pore distribution in the foam. A decrease in the pressure, temperature and saturation time leads to the formation of structures with a high mechanical strength and a high degree of crystallinity. Among the combinations of process parameters tested in this work, the optimum conditions for the production of solid foams with the desired properties for biomedical purposes are pressure 18 MPa, temperature 70 °C and time 1 h. The addition of blowing agents leads to changes in the properties of the obtained solid foams. The addition of a porogen unit leads to a decrease in the pore size and an increase in the density of the nucleation sites, mechanical strength and degree of crystallinity of the obtained porous structure compared to the foam made of pure PCL.

The type of porogen has a significant effect on the properties of the obtained solid foams. Under the same operating conditions of the foaming process and porogen concentration, the most significant yet beneficial changes were obtained by the addition of graphene oxide and hydroxyapatite. The size of the hydroxyapatite grains has a significant effect on the values of the morphology parameters of solid foams. For grains at the microscale, an increase in concentration leads to an increase in the pore diameter and a decrease in the pores density, while at the nanoscale the opposite relationship was obtained. Taking into account the morphological features and mechanical properties required in biomedical applications of solid foams, a composite containing 5% hydroxyapatite of 0.2% graphene oxide is recommended. 

In this work, the novel application of various types of porogens—hydroxyapatite, nanocellulose, carboxymethylcellulose, and graphene oxide—was tested in order to modify the properties and improve the quality of PCL-based solid foams, increasing their usefulness in specialized practical applications. For the first time, the potential of the use of micro- and nanosized porogens to influence the process of PCL foaming using scCO_2_ was demonstrated. By the selection of the proper process conditions, as well as the type and amount of the porogen, composite solid foams with smaller pores and higher mechanical strength—when compared to foams produced with pure PCL—can be obtained. The presented composite solid foams are potentially suitable for application in tissue engineering; however, their biological properties, and especially their biocompatibility, have to be assessed.

## Figures and Tables

**Table 1:** Characteristics of the raw materials used in the production of the composite materials.

Chemical Substance	Form	Particle Size	Supplier	Characteristics
Poly (ɛ-caprolactone)(PCL)	Granules	~3 mm	Sigma Aldrich (Poznan, Poland)	Solid granulate with a molar mass of 80,000, a relatively low melting point of 60 °C and a density of 1.145 g/mL
Hydroxyapatite (µHA)	Powder	10 µm ± 2 µm		Synthetic powder, specific surface area ≥10 m^2^/g
Hydroxyapatite (nHA)		<200 nm		Synthetic nanopowder with a purity ≥97%, mp 1100 °C, specific surface area 9.4 m^2^/g
Carboxymethylcellulose (µCMC)		10–16 µm		Solid white in fibrous form, CMC soluble in water at 25 °C at a concentration of 4% with a low viscosity of 50–200 mPas, used as a filler, with good wettability
Nanocellulose (nC)		Particles 10–20 nm wide, 50–400 nm long	CelluloseLab (Fredericton, Canada)	Plant material, preparation by acid hydrolysis, crystallinity coefficient >70%
Graphene oxide (nGO)	Suspension in ethyl alcohol	10 layers of flake graphene, each layer from several nm to several hundred µm, with 0.4–0.9 nm between them	Nanomaterials (Warsaw, Poland)	Flake, multi-layered graphene oxide in suspension, odorless, suspensions of varying light to dark brown color, flash point 180–220 °C, pH 5–7

**Table 2:** Detailed data on the conditions of the implementation of the process of producing solid foams using composite materials.

**Pure PCL**									
**M [g]**	**Raw material**		**P_sat_ [MPa]**	**T_sat_ [°C]**	**t_sat_ [h]**	**P_foam_ [MPa]**	**T_foam_ [°C]**	**t_foam_ [h]**	**D [MPa/h]**
3	PCL		9–18	50–100	0.5–6	9–18	25	0.08–0.5	60 →∞
**Composite Material**									
**M [g]**	**Porogen**	**C [%(*w/w*)]**	**Psat [MPa]**	**T_sat_ [°C]**	**t_sat_ [h]**	**P_foam_ [MPa]**	**T_foam_ [°C]**	**t_foam_ [h]**	**D [MPa/h]**
3	µHA	5–20	9–18	50–100	1–4	9–18	25	0.5	→∞
	nHA	1–12	9–18		0.5–4	9–18			
	µCMC								
	nC								
	nGO	0.2–1.5							

## Data Availability

Not applicable.
